# Crystal structure reinvestigation of silver(I) fluoride, AgF

**DOI:** 10.1107/S2414314623000184

**Published:** 2023-01-24

**Authors:** Matic Lozinšek, Matic Belak Vivod, Mirela Dragomir

**Affiliations:** a Jožef Stefan Institute, Jamova cesta 39, 1000 Ljubljana, Slovenia; b Jožef Stefan International Postgraduate School, Jamova cesta 39, 1000, Ljubljana, Slovenia; Vienna University of Technology, Austria

**Keywords:** silver(I) fluoride, silver fluorides, crystal structure, single-crystal X-ray diffraction

## Abstract

A crystal structure reinvestigation of AgF based on a low-temperature high-resolution single-crystal X-ray diffraction data is reported. Silver(I) fluoride crystallizes in the rock salt structure type (*Fm*  *m*) with a unit-cell parameter of 4.92171 (14) Å at 100 K, resulting in an Ag—F bond length of 2.46085 (7) Å.

## Abstract 2

The rock salt-type crystal structure of AgF was re-examined from a high-resolution, low-temperature single-crystal X-ray diffraction data set.

## Graphical abstract

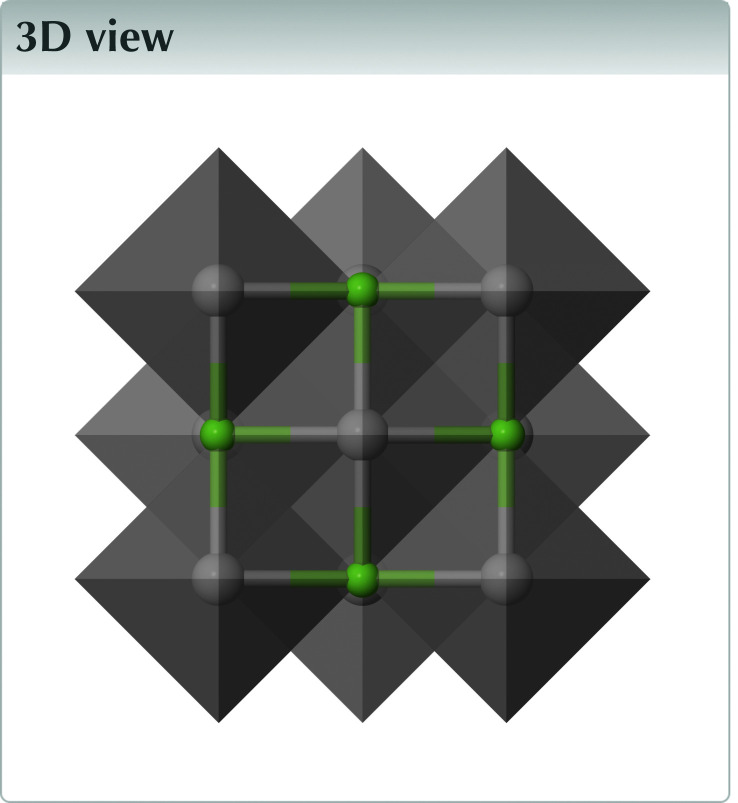

## Structure description

Crystal structure data of the following binary silver fluorides can be retrieved from the Inorganic Crystal Structure Database (ICSD; Bergerhoff *et al.*, 1983 ▸; Zagorac *et al.*, 2019 ▸): Ag_2_F, AgF, AgF_2_, AgF_3_, Ag_2_F_5_, and Ag_3_F_8_ (Table 1 ▸).

The crystal structure of silver subfluoride, Ag_2_F, was elucidated from powder and single-crystal X-ray diffraction data (Ott & Seyfarth, 1928 ▸; Terrey & Diamond, 1928 ▸; Argay & Náray-Szabó, 1966 ▸) as well as studied by powder neutron diffraction measurements from room temperature to 20 K (Williams, 1989 ▸). Silver(I) fluoride, AgF, has been investigated at ambient conditions only by powder X-ray diffraction (Ott, 1926 ▸; Bottger, & Geddes, 1972 ▸). The high-pressure structural behavior of AgF was thoroughly studied by powder X-ray diffraction (Halleck *et al.*, 1972 ▸; Jamieson *et al.*, 1975 ▸), powder neutron diffraction experiments to 6.5 GPa (Hull & Berastegui, 1998 ▸), and by synchrotron powder X-ray diffraction measurements up to 39 GPa (Grzelak *et al.*, 2017*a* ▸). Silver(II) fluoride, AgF_2_, was studied by powder X-ray diffraction (Ruff & Giese, 1934 ▸; Charpin *et al.*, 1966 ▸; Baturina *et al.*, 1967 ▸; Kiselev *et al.*, 1988 ▸) and its crystal structure determined from single-crystal X-ray diffraction data (Jesih *et al.*, 1990 ▸), as well as by powder neutron diffraction (Charpin *et al.*, 1970 ▸; Fischer *et al.*, 1971 ▸). Moreover, the high-pressure structural behavior of AgF_2_ was explored employing synchrotron X-ray diffraction (Grzelak *et al.*, 2017*a* ▸,*b* ▸). The crystal structure of silver(III) fluoride, AgF_3_, was refined from powder neutron diffraction data and synchrotron powder X-ray diffraction data was also measured (Žemva *et al.*, 1991 ▸). A mixed-valence silver(II,III) fluoride Ag_2_F_5_, or Ag^II^F[Ag^III^F_4_], was structurally characterized by single-crystal X-ray diffraction (Fischer & Müller, 2002 ▸), whereas the crystal structure of Ag_3_F_8_, or Ag^II^[Ag^III^F_4_]_2_, was determined by synchrotron powder X-ray diffraction (Graudejus *et al.*, 2000 ▸). Two recent reports explored the pressure–composition phase diagram of binary silver fluorides by theoretical methods (Kurzydłowski *et al.*, 2021 ▸; Rybin *et al.*, 2022 ▸).

**Figure 1:**
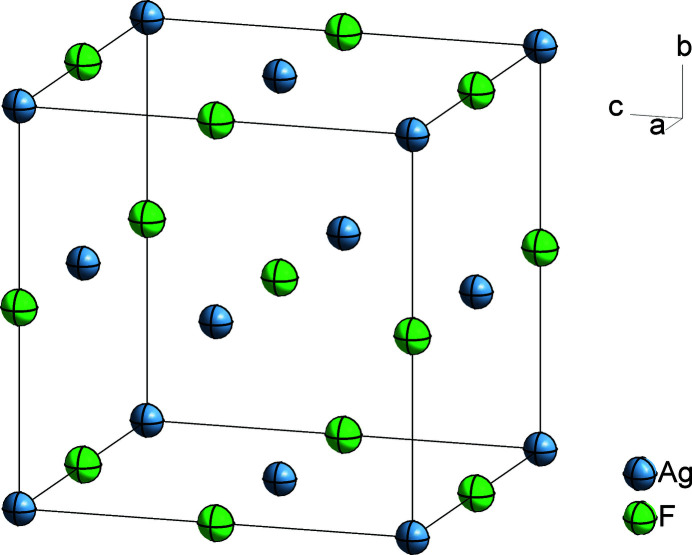
The rock salt-type structure of AgF. Displacement ellipsoids are drawn at the 50% probability level.

Herein, a low-temperature high-resolution (0.54 Å) single-crystal X-ray diffraction measurement of AgF (rock salt structure type, *Fm* 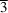 *m*) is reported (Fig. 1 ▸). The unit-cell parameter (Table 2 ▸) is in good agreement with the previously reported room-temperature value of 4.936 (1) Å (Bottger & Geddes, 1972 ▸). The Ag—F bond length determined from the current low-temperature data is 2.46085 (7) Å.

## Synthesis and crystallization

Agglomerated single-crystals with typical dimensions of ∼50 µm were recovered from a solid-state reaction (*T* = 310 °C) where AgF (Thermo Scientific, 99+%) was used as a starting material. A small amount of sample was placed onto a watch glass and covered with a protective layer of perfluoro­deca­lin (Fluorochem, 96.0%, *cis* and *trans*) inside a nitro­gen-filled glovebox (Vigor, H_2_O < 0.1 ppm). The sample was examined under a polarizing microscope outside the glovebox, and selected crystals were mounted on a MiTeGen Dual Thickness MicroLoops with the aid of Baysilone-Paste (Bayer-Silicone, mittelviskos).

## Refinement

Crystal data, data collection and structure refinement details are summarized in Table 2 ▸.

## Supplementary Material

Crystal structure: contains datablock(s) global, I. DOI: 10.1107/S2414314623000184/wm4179sup1.cif

Structure factors: contains datablock(s) I. DOI: 10.1107/S2414314623000184/wm4179Isup2.hkl

CCDC reference: 2235315

Additional supporting information:  crystallographic information; 3D view; checkCIF report

## Figures and Tables

**Table 1:** Crystal structure data of binary silver fluorides reported in the ICSD database (only one entry for each crystalline phase is given) PND = powder neutron diffraction; PXRD = powder X-ray diffraction; SCXRD = single-crystal X-ray diffraction.

Compound	Space group	Unit-cell parameters	Method, conditions	Reference
Ag_2_F	*P* 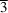 *m*1	*a* = 2.99877 (5) Å, *c* = 5.6950 (2) Å	PND, 300 K	Williams (1989 ▸)
AgF	*Fm* 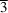 *m*	*a* = 4.92 Å	PXRD	Ott (1926 ▸)
AgF_2_	*P*2_1_/*n*	*a* = 3.34 Å, *b* = 4.57 Å, *c* = 4.65 Å, *β* = 84.5°	PXRD, 123 K / 195 K	Baturina *et al.* (1967 ▸)
AgF_2_	*Pbca*	*a* = 5.568 (1) Å, *b* = 5.831 (1) Å, *c* = 5.101 (1) Å	SCXRD	Jesih *et al.* (1990 ▸)
AgF_2_	*Pbcn*	*a* = 5.476 (10) Å, *b* = 8.331 (15) Å, *c* = 5.787 (7) Å	Synchrotron PXRD, 14.8 GPa	Grzelak *et al.* (2017*b* ▸)
AgF_2_	*Pca*2_1_	*a* = 5.475 (7) Å, *b* = 4.704 (6) Å, *c* = 5.564 (6) Å	Synchrotron PXRD, 10 GPa	Grzelak *et al.* (2017*a* ▸)
AgF_3_	*P*6_1_22	*a* = 5.0782 (2) Å, *c* = 15.4523 (8) Å	PND	Žemva *et al.* (1991 ▸)
Ag_2_F_5_	*P* 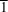	*a* = 4.999 (2) Å, *b* = 11.087 (5) Å, *c* = 7.357 (3) Å, *α* = 90.05 (3)°, *β* = 106.54 (4)°, *γ* = 90.18 (4)°	SCXRD	Fischer & Müller (2002 ▸)
Ag_3_F_8_	*P*2_1_/*n*	*a* = 5.04664 (8) Å, *b* = 11.0542 (2) Å, *c* = 5.44914 (9) Å, *β* = 97.170 (2)°	Synchrotron PXRD, 299 K	Graudejus *et al.* (2000 ▸)

**Table 2:** Experimental details

Crystal data	
Chemical formula	AgF
*M*_r_	126.87
Crystal system, space group	Cubic, *F* *m* 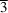 *m*
Temperature (K)	100
*a* (Å)	4.92171 (14)
*V* (Å^3^)	119.22 (1)
*Z*	4
Radiation type	Ag *K*α, λ = 0.56087 Å
μ (mm^−1^)	8.51
Crystal size (mm)	0.06 × 0.05 × 0.05

Data collection	
Diffractometer	XtaLAB Synergy-S, Dualflex, Eiger2 R CdTe 1M
Absorption correction	Multi-scan (*CrysAlis PRO*; Rigaku OD, 2022 ▸)
*T*_min_, *T*_max_	0.724, 1.000
No. of measured, independent and observed [*I* > 2σ(*I*)] reflections	730, 37, 37
*R*_int_	0.050
(sin θ/λ)_max_ (Å^−1^)	0.926

Refinement	
*R*[*F* ^2^ > 2σ(*F* ^2^)], *wR*(*F* ^2^), *S*	0.011, 0.020, 1.28
No. of reflections	37
No. of parameters	3
Δρ_max_, Δρ_min_ (e Å^−3^)	0.59, −0.55

Computer programs: *CrysAlis PRO* (Rigaku OD, 2022 ▸), *OLEX2.solve* and *OLEX2* (Dolomanov *et al.*, 2009 ▸), *SHELXL* (Sheldrick, 2015 ▸), *DIAMOND* (Brandenburg, 2005 ▸) and *publCIF* (Westrip, 2010 ▸).

## full crystallographic data

### Crystal data

**Table:**

AgF	Ag *K*α radiation, λ = 0.56087 Å
*M**_r_* = 126.87	Cell parameters from 689 reflections
Cubic, *F**m*3*m*	θ = 5.7–31.2°
*a* = 4.92171 (14) Å	µ = 8.51 mm^−^^1^
*V* = 119.22 (1) Å^3^	*T* = 100 K
*Z* = 4	Irregular, colourless
*F*(000) = 224	0.06 × 0.05 × 0.05 mm
*D*_x_ = 7.068 Mg m^−^^3^	

### Data collection

**Table:**

XtaLAB Synergy-S, Dualflex, Eiger2 R CdTe 1M diffractometer	37 independent reflections
Radiation source: micro-focus sealed X-ray tube, PhotonJet (Ag) X-ray Source	37 reflections with *I* > 2σ(*I*)
Mirror monochromator	*R*_int_ = 0.050
Detector resolution: 13.3333 pixels mm^-1^	θ_max_ = 31.3°, θ_min_ = 5.7°
ω scans	*h* = −9→8
Absorption correction: multi-scan (CrysAlisPro; Rigaku OD, 2022)	*k* = −9→8
*T*_min_ = 0.724, *T*_max_ = 1.000	*l* = −8→8
730 measured reflections	

### Refinement

**Table:**

Refinement on *F*^2^	0 restraints
Least-squares matrix: full	Primary atom site location: iterative
*R*[*F*^2^ > 2σ(*F*^2^)] = 0.011	*w* = 1/[σ^2^(*F*_o_^2^) + 0.2914*P*] where *P* = (*F*_o_^2^ + 2*F*_c_^2^)/3
*wR*(*F*^2^) = 0.020	(Δ/σ)_max_ < 0.001
*S* = 1.28	Δρ_max_ = 0.59 e Å^−^^3^
37 reflections	Δρ_min_ = −0.55 e Å^−^^3^
3 parameters	

### Special details

**Table:**

Geometry. All esds (except the esd in the dihedral angle between two l.s. planes) are estimated using the full covariance matrix. The cell esds are taken into account individually in the estimation of esds in distances, angles and torsion angles; correlations between esds in cell parameters are only used when they are defined by crystal symmetry. An approximate (isotropic) treatment of cell esds is used for estimating esds involving l.s. planes.

### Fractional atomic coordinates and isotropic or equivalent isotropic displacement parameters (Å^2^)

**Table:**

	*x*	*y*	*z*	*U*_iso_*/*U*_eq_	
Ag1	0.000000	0.000000	0.000000	0.01650 (12)	
F1	0.500000	0.000000	0.000000	0.0199 (8)	

### Atomic displacement parameters (Å^2^)

**Table:**

	*U*^11^	*U*^22^	*U*^33^	*U*^12^	*U*^13^	*U*^23^
Ag1	0.01650 (12)	0.01650 (12)	0.01650 (12)	0.000	0.000	0.000
F1	0.0199 (8)	0.0199 (8)	0.0199 (8)	0.000	0.000	0.000

### Geometric parameters (Å, º)

**Table:**

Ag1—F1^i^	2.4609 (1)	Ag1—F1^iii^	2.4609 (1)
Ag1—F1^ii^	2.4609 (1)	Ag1—F1^iv^	2.4609 (1)
Ag1—F1	2.4609 (1)	Ag1—F1^v^	2.4609 (1)
			
F1^i^—Ag1—F1	90.0	Ag1^vi^—F1—Ag1	90.0
F1^v^—Ag1—F1^iii^	180.0	Ag1^vii^—F1—Ag1^viii^	180.0
F1^v^—Ag1—F1^ii^	90.0	Ag1^vii^—F1—Ag1^ix^	90.0
F1^iv^—Ag1—F1^iii^	90.0	Ag1^x^—F1—Ag1^viii^	90.0
F1—Ag1—F1^ii^	90.0	Ag1—F1—Ag1^ix^	90.0
F1^iv^—Ag1—F1^ii^	90.0	Ag1^x^—F1—Ag1^ix^	90.0
F1^i^—Ag1—F1^v^	90.0	Ag1—F1—Ag1^viii^	90.0
F1^i^—Ag1—F1^iv^	90.0	Ag1^vi^—F1—Ag1^x^	90.0
F1—Ag1—F1^v^	90.0	Ag1—F1—Ag1^vii^	90.0
F1^i^—Ag1—F1^ii^	180.0	Ag1^vi^—F1—Ag1^ix^	180.0
F1^iv^—Ag1—F1^v^	90.0	Ag1^x^—F1—Ag1^vii^	90.0
F1—Ag1—F1^iv^	180.0	Ag1—F1—Ag1^x^	180.0
F1^i^—Ag1—F1^iii^	90.0	Ag1^vi^—F1—Ag1^viii^	90.0
F1—Ag1—F1^iii^	90.0	Ag1^viii^—F1—Ag1^ix^	90.0
F1^iii^—Ag1—F1^ii^	90.0	Ag1^vi^—F1—Ag1^vii^	90.0

Symmetry codes: (i) *x*−1/2, *y*+1/2, *z*; (ii) *x*−1/2, *y*−1/2, *z*; (iii) *x*−1/2, *y*, *z*+1/2; (iv) *x*−1, *y*, *z*; (v) *x*−1/2, *y*, *z*−1/2; (vi) *x*+1/2, *y*+1/2, *z*; (vii) *x*+1/2, *y*, *z*−1/2; (viii) *x*+1/2, *y*, *z*+1/2; (ix) *x*+1/2, *y*−1/2, *z*; (x) *x*+1, *y*, *z*.
